# Optical Coherence Tomography Angiography in Type 1 Diabetes Mellitus. Report 5: Cardiovascular Risk

**DOI:** 10.3390/biomedicines14010153

**Published:** 2026-01-11

**Authors:** Josep Rosinés-Fonoll, Ruben Martin-Pinardel, Sonia Marias-Perez, Xavier Suarez-Valero, Silvia Feu-Basilio, Sara Marín-Martinez, Carolina Bernal-Morales, Rafael Castro-Dominguez, Andrea Mendez-Mourelle, Cristian Oliva, Irene Vila, Teresa Hernández, Irene Vinagre, Manel Mateu-Salat, Emilio Ortega, Marga Gimenez, Javier Zarranz-Ventura

**Affiliations:** 1 Institut Clínic d’Oftalmologia (ICOF), Hospital Clínic, 08028 Barcelona, Spain; rosines@clinic.cat (J.R.-F.); smarias@clinic.cat (S.M.-P.); xavi.suarez96@gmail.com (X.S.-V.); sfeu@clinic.cat (S.F.-B.); samarin@clinic.cat (S.M.-M.); bernal@clinic.cat (C.B.-M.); rcastrod@recerca.clinic.cat (R.C.-D.); anmendez@clinic.cat (A.M.-M.); coliva@clinic.cat (C.O.); irvila@clinic.cat (I.V.); tessa.hrndz@gmail.com (T.H.); 2 Department of Surgery and Medical-Surgical Specialties, Faculty of Medicine and Health Sciences, Universitat de Barcelona, 08036 Barcelona, Spain; rbnmartinpinardel@gmail.com; 3 Fundació de Recerca Clínic Barcelona-Institut d’Investigacions Biomèdiques August Pi i Sunyer (IDIBAPS), 08036 Barcelona, Spain; ivinagre@clinic.cat (I.V.); mamateu@clinic.cat (M.M.-S.); eortega1@clinic.cat (E.O.); gimenez@clinic.cat (M.G.); 4 Diabetes Unit, Institut Clínic de Malalties Digestives i Metabòliques (ICMDM), Hospital Clínic, 08036 Barcelona, Spain; 5 Department of Medicine, Faculty of Medicine and Health Sciences, Universitat de Barcelona, 08036 Barcelona, Spain; 6 Centro de Investigación Biomédica en Red de la Fisiopatología de la Obesidad y Nutrición (CIBEROBN), 28029 Madrid, Spain

**Keywords:** diabetic retinopathy, cardiovascular risk assessment, cardiovascular risk predictors, vessel density, perfusion density, foveal avascular zone, central macular thickness, retinal nerve fiber layer, optical coherence tomography, optical coherence tomography angiography

## Abstract

**Objectives**: This study aimed to investigate the association between optical coherence tomography angiography (OCTA) parameters and cardiovascular (CV) risk scores in individuals with type 1 diabetes (T1D). **Methods**: A cross-sectional analysis of a large-scale prospective OCTA trial cohort (ClinicalTrials.gov NCT03422965) was performed. Demographic, systemic, and ocular data—including OCTA imaging—were collected. T1D participants were stratified into three CV risk categories: moderate (MR), high (HR), and very high risk (VHR). Individualized predictions for fatal and non-fatal CV events at 5 and 10 years were calculated using the STENO T1 Risk Engine calculator. **Results**: A total of 501 individuals (1 eye/patient; 397 T1D, 104 controls) were included. Subjects with MR (*n* = 37), HR (*n* = 152) and VHR (*n* = 208) exhibited significantly reduced vessel density (VD) (20.9 ± 1.3 vs. 20.2 ± 1.6 vs. 19.3 ± 1.8 mm^−1^, *p* < 0.05), perfusion density (PD) (0.37 ± 0.02 vs. 0.36 ± 0.02 vs. 0.35 ± 0.02%, *p* < 0.05) and foveal avascular zone circularity (0.69 ± 0.06 vs. 0.65 ± 0.07 vs. 0.63 ± 0.09, *p* < 0.05). Statistically significant negative correlations were observed between CV risk and OCTA parameters including VD, PD, and retinal nerve fiber layer thickness, while central macular thickness (CMT) showed a positive correlation (*p* < 0.05). Notably, CMT was significantly associated with 5-year CV risk. **Conclusions**: OCTA-derived metrics, particularly reduced retinal VD and PD, are associated with elevated CV risk scores in T1D patients. These findings suggest that OCTA may serve as a valuable non-invasive tool for identifying individuals with increased CV risk scores.

## 1. Introduction

Cardiovascular (CV) disease remains a leading cause of global mortality and morbidity, accounting for an estimated 17.8 million deaths in 2017 [1]. Stratification of patients based on modifiable risk factors such as tobacco and alcohol use, obesity, physical inactivity, or hyperlipidaemia, and the presence of diabetes mellitus (DM) is critical for effective prevention strategies. The identification and management of these risk factors are essential to mitigate the incidence of CV disease and its associated complications [2]. DM has emerged as one of the major public health challenges of the 21st century, with type 1 diabetes (T1D) affecting an estimated 8–9 million individuals worldwide [3]. Numerous studies have demonstrated a strong association between DM and CV events, particularly in individuals with type 2 diabetes (T2D). However, the pathophysiological mechanisms linking CV events, risk factors, and T1D remain poorly understood [4].

Optical Coherence Tomography Angiography (OCTA) is an innovative, rapid, and non-invasive imaging modality based on Optical Coherence Tomography (OCT) technology that enables the reconstruction of three-dimensional representations of the retinal microvascular architecture. This technique provides in situ, high-resolution visualization of distinct retinal vascular layers and, critically, facilitates objective and quantitative assessment of vascular morphological features [5]. Quantitative OCTA metrics, including reduced vessel density (VD), paramacular non-perfusion zones, and alterations in the foveal avascular zone (FAZ), have been reported in diabetic retinopathy (DR) [6], even preceding the onset of clinically detectable signs [7]. Multiple studies have sought to identify DR biomarkers derived from OCTA-generated quantitative data [8,9]. Moreover, emerging evidence suggests that retinal microvascular alterations detected via OCTA may serve as early indicators of systemic CV disease [10,11,12].

CV risk assessment is a key strategy in the prevention and management of CV disease, typically performed using predictive models based on clinical risk factors [13,14]. Currently, no specific and widely implemented CV risk prediction algorithms are available for individuals with T1D in routine clinical care. Existing models, largely derived from T2D or general population cohorts, have demonstrated limited predictive accuracy in T1D populations and tend to underestimate risk [15]. Despite these limitations, the European Society of Cardiology (ESC) clinical practice guidelines for CV disease prevention recommend applying the same risk stratification framework to patients with T1D as is used for those with T2D [2]. In response to this gap, novel tools and models have recently been developed to improve CV risk prediction specifically in T1D patients [16,17].

The STENO Type 1 Risk Engine is a CV risk prediction model designed to estimate the 5- and 10-year probability of both fatal and non-fatal CV events in individuals with T1D. It incorporates ten clinical variables: age, sex, duration of diabetes, glycated hemoglobin (HbA1c), blood pressure, low-density lipoprotein (LDL) cholesterol, estimated glomerular filtration rate, albuminuria, smoking status, and physical activity. This tool has been clinically validated in a cohort of 4306 adult persons with T1D without prior CV events [17]. According to the ESC guidelines, most people with T1D are classified into high or very high CV risk categories. This classification entails stringent therapeutic targets for blood pressure, lipid levels, and antiplatelet therapy, posing challenges for the standardization of generalized risk profiles [18].

This study aims to investigate potential associations between retinal microvascular parameters, as measured by OCTA, and CV risk stratification based on standardized prediction scales in a large cohort of patients with T1D enrolled in a prospective OCTA trial. The primary objective is to assess the utility of OCTA as a rapid, non-invasive, and objective tool for evaluating CV risk in T1D patients. Such an approach may offer valuable insights for systemic risk assessment and contribute to improved clinical management strategies in this population. This study is the Report 5 of a series of cross-sectional analyses aimed at investigating relationships between OCTA metrics and diabetic retinopathy (report 1), diabetic kidney disease (report 2), the impact of scan field (report 3) and glycemic control (report 4) in a prospective cohort of T1D patients generated in a previous prospective trial.

## 2. Materials and Methods

### 2.1. Study Design and Study Protocol

This cross-sectional, exploratory study involved the prospective acquisition of OCTA images alongside ocular and systemic clinical data from a large cohort of people with T1D. The detailed study protocol has been previously published [19]. Ethical approval was obtained from the Institutional Review Board of Hospital Clínic of Barcelona (HCB/2016/0216, approval date 16 December 2016), and the original study was registered on ClinicalTrials.gov (Identifier: NCT03422965, registered 8 May 2017). All participants provided written informed consent prior to enrollment.

### 2.2. Inclusion and Exclusion Criteria

People with T1D were recruited from the Diabetes Unit and subsequently referred to the Ophthalmology Department for a comprehensive ophthalmologic evaluation. Control subjects were enrolled through outreach campaigns coordinated by the hospital’s communication department. Exclusion criteria included: age under 18 years; presence of ocular opacities or ocular comorbidities (e.g., macular edema, prior ophthalmic surgery, laser treatment, intravitreal therapy, glaucoma, amblyopia, retinal artery or vein occlusion, uveitis, or extreme axial length); inability to complete the ophthalmologic examination adequately; and refusal to provide written informed consent.

### 2.3. Ocular and Systemic Data

Systemic data collected included general demographic and clinical characteristics (e.g., age, sex, smoking status, blood pressure, and body mass index), as well as diabetes-related variables (e.g., duration of diabetes, presence of macrovascular complications, and insulin requirements). Laboratory assessments comprised HbA1c, total cholesterol, LDL cholesterol, high-density lipoprotein (HDL) cholesterol, triglycerides (TG), hemoglobin, and platelet count. Ocular data included best-corrected visual acuity (BCVA), slit-lamp biomicroscopy, spherical equivalent, fundus examination, and ocular biometry (IOL Master; Carl Zeiss Meditec, Dublin, CA, USA). DR severity was graded according to the International Clinical Diabetic Retinopathy Disease Severity Scale [20].

### 2.4. Structural OCT and OCTA Imaging Protocols

All OCT and OCTA images were acquired using the Cirrus HD-OCT system (Carl Zeiss Meditec, Dublin, CA, USA). Structural OCT protocols included macular cube scans (512 × 128 pixels) and optic nerve head scans (212 × 212 pixels). OCTA scans were performed using 3 × 3 mm cubes centered on the fovea. Image quality was assessed for all OCT and OCTA scans, and those presenting artifacts, segmentation errors, or a signal strength index (SSI) below 7 were excluded from analysis. Structural OCT parameters included central macular thickness (CMT), macular volume (MV), average macular thickness (AMT), and retinal nerve fiber layer (RNFL) thickness. OCTA metrics were quantified using the built-in AngioPlex Metrix software (version 11.0.0) (Carl Zeiss Meditec, Dublin, CA, USA), focusing on the superficial capillary plexus (SCP), defined by the boundaries of the internal limiting membrane and the inner plexiform layer. OCTA measurements included vessel density (VD, mm^−1^), perfusion density (PD), and foveal avascular zone (FAZ) metrics: area (FAZa, mm^2^), perimeter (FAZp, mm), and circularity (FAZc, %). No manual adjustments to segmentation slabs were performed.

### 2.5. Cardiovascular Risk and STENO Type 1 Risk Engine Stratification Protocol

All patients were stratified according to two CV risk assessment tools: the ESC classification and the STENO Type 1 Risk Engine [2,17]. The ESC system categorizes individuals into moderate (MR), high (HR), and very high (VHR) CV risk groups. The STENO Type 1 Risk Engine estimates the 5- and 10-year risk of CV events based on ten clinical variables: age, sex, duration of diabetes, HbA1c, blood pressure, LDL cholesterol, glomerular filtration rate, albuminuria, smoking status, and physical activity. The resulting score is interpreted according to the National Institute for Health and Care Excellence (NICE) guidelines: <10% (low risk), 10–20% (moderate risk), and >20% (high risk).

### 2.6. Statistical Analysis

Quantitative variables were summarized using mean, standard deviation (SD), median, and interquartile range (Q1, Q3). Qualitative variables were described using absolute frequencies and percentages. The normality of data distributions was assessed using the Shapiro–Wilk test. Formal sample size was not calculated as data was generated in a previous OCTA trial. Group comparisons were performed using ANOVA, Kruskal–Wallis, Chi-square tests and Fisher’s exact tests, as appropriate. Power analysis for intergroup comparisons is detailed in Supplementary File S1. Pairwise comparisons were conducted using *t*-test or the Mann–Whitney U test. Both unadjusted and adjusted *p*-values (multiple linear regression model adjusted for age, sex, SSI, axial length, CMT, and duration of diabetes) were reported. A regression model was applied to assess correlations between variables, including a robust regression to check the possible effects of outliers. The variables selected to adjust the regression models were driven by clinical knowledge rather than data-driven, to avoid dataset-specific conclusions and allow greater generalizability. No correction for multiple testing was performed. Statistical significance was defined as a *p*-value < 0.05. All analyses were conducted using R Studio (version 4.1.2).

## 3. Results

**Figure 1:**
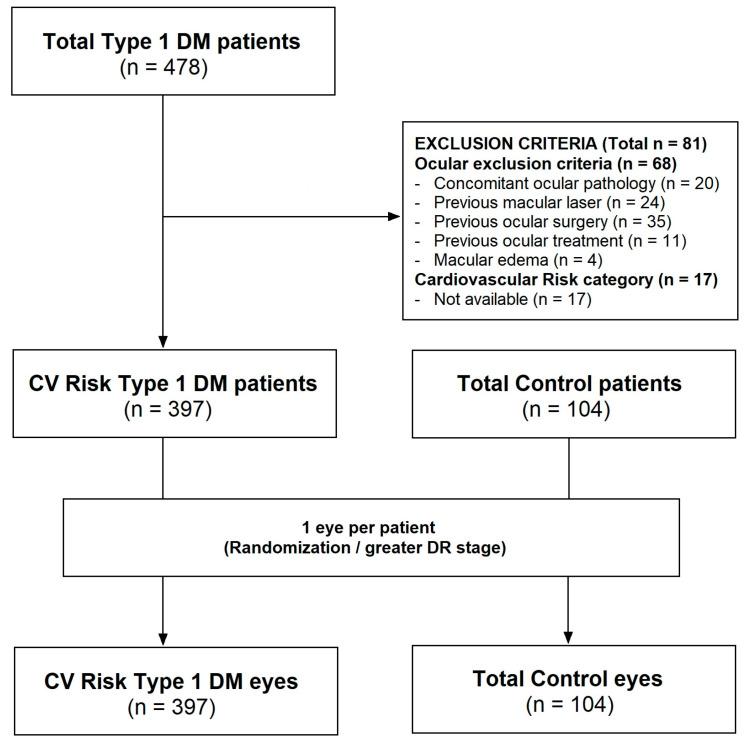
CONSORT-style flow diagram detailing the inclusion and exclusion of patients and eyes for each OCTA analysis.

A consolidated standard of reporting trials (CONSORT)-style flow diagram detailing the inclusion and exclusion of patients and eyes for each OCTA analysis is provided in Figure 1. Data from 501 individuals were initially evaluated. To minimize the risk of bilaterality bias, only one eye per participant was included in the analysis (1 patient/1 eye; *n* = 501 eyes; 397 with T1D and 104 controls). A detailed summary of excluded images due to SSI < 7 per groups is presented in Supplementary File S2. After applying exclusion criteria, a total of 485 patients were retained for final analysis.

### 3.1. Baseline Characteristics and Study Groups

The baseline characteristics of the study cohort are summarized in Table 1, Table 2 and Table 3. Patients classified as MR and HR were significantly younger than control subjects (28.1 ± 5.83 vs. 38.18 ± 12.06 vs. 43.35 ± 14.25 years; *p* < 0.001). Patients in the VHR group exhibited a significantly higher body mass index (BMI) compared to controls (25.42 ± 3.88 vs. 23.64 ± 3.50; *p* < 0.001). Within the T1D subgroup, VHR patients had a significantly longer duration of DM compared to MR and HR groups (25.6 vs. 15.8 vs. 5.58 years; *p* < 0.001).

### 3.2. Cardiovascular Risk Stratification Groups and OCTA Metrics

**Figure 2:**
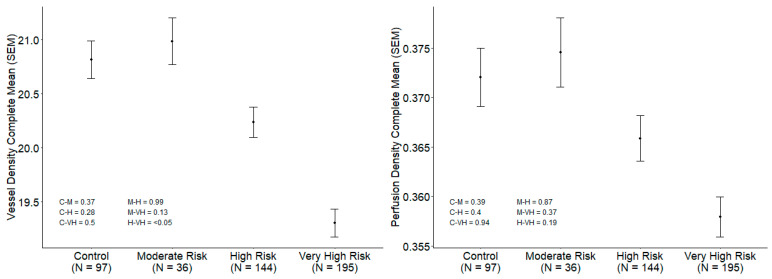
Subgroup analysis based on CV risk stratification. Vessel density and perfusion density (Control, MR, HR and VHR).

**Figure 3:**
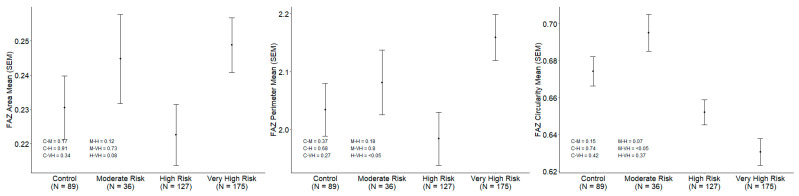
Subgroup analysis based on CV risk stratification. Foveal avascular zone parameters (Control, MR, HR and VHR).

Subgroup analysis based on CV risk stratification is summarized in Table 4 and represented in Figure 2 and Figure 3. VD was significantly lower in the VHR and HR groups compared to the MR and control groups (19.30 ± 1.81 vs. 20.24 ± 1.67 vs. 20.99 ± 1.30 vs. 20.82 ± 1.73 mm^−1^; *p* < 0.001). After adjusting for age, sex, axial length, SSI, DM duration and CMT the differences remained statistically significant in the VHR group compared to MR and HR (*p* = 0.04) (Figure 2). The VHR group also showed significantly greater FAZa and FAZp compared to the HR group (0.249 ± 0.105 vs. 0.223 ± 0.101 mm^2^ and 2.159 ± 0.526 vs. 1.984 ± 0.514 mm, *p* = 0.03 and *p* = 0.004, respectively) (Figure 3). CMT was significantly lower in the MR group compared to HR and VHR (256.95 ± 16.35 vs. 264.03 ± 21.17 vs. 264.22 ± 22.42 µm; *p* = 0.03 and *p* = 0.02, respectively). RNFL thickness was also significantly lower in MR compared to HR, VHR, and controls (93.63 ± 10.06 vs. 96.22 ± 10.29 vs. 96.88 ± 11.03 vs. 96.59 ± 9.05 µm; *p* = 0.03). Stability of the results was evaluated using bootstrapping and multivariate regression (ANCOVA), presented in Supplementary File S3.

### 3.3. Correlations Between STENO-T1 Risk Score and Structural OCT and OCTA Parameters

**Figure 4:**
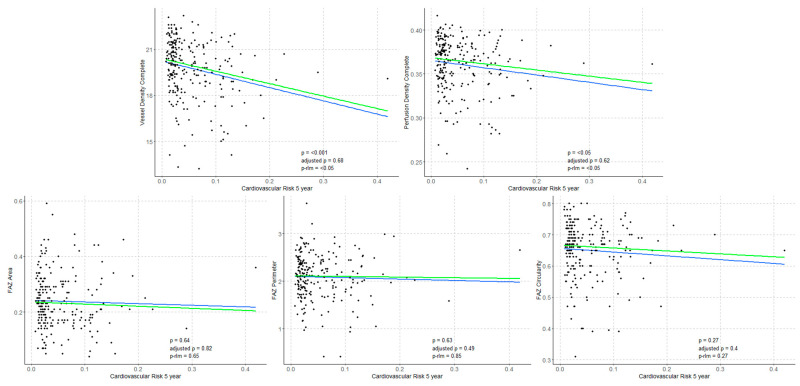
Regression analyses between OCTA parameters (VD, PD, FAZa, FAZp, FAZc) with CV risk scores for fatal and non-fatal events at 5 years. Blue: regression line (p), green: robust regression line (sensitivity analysis, p-rlm).

**Figure 5:**
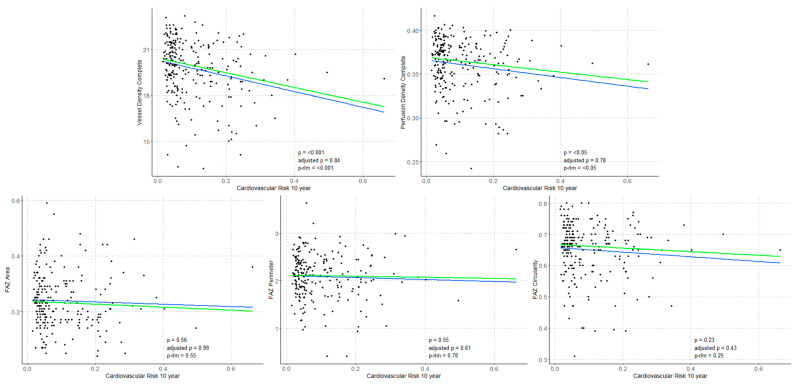
Regression analyses between OCTA parameters (VD, PD, FAZa, FAZp, FAZc) with CV risk scores for fatal and non-fatal events at 10 years. Blue: regression line (p), green: robust regression line (sensitivity analysis, p-rlm).

Regression analyses were conducted to evaluate the associations between OCTA parameters (VD, PD, FAZa, FAZp and FAZc) and structural OCT metrics (CRT, MV, AMT and RNFL) with CV risk scores for fatal and non-fatal events at 5 and 10 years. Significant inverse associations were found between VD, PD, and RNFL thickness and CV risk scores at both 5 and 10 years (*p* < 0.001, *p* < 0.05, and *p* < 0.001, respectively). In contrast, CMT showed a significant positive correlation with CV risk at both time points (*p* < 0.001). These findings are illustrated in Figure 4 and Figure 5.

## 4. Discussion

This study identifies significant associations between CV risk profiles and OCTA metrics in a large cohort of people with T1D and controls. Our findings demonstrate that HR and VHR groups, as defined by ESC guidelines, exhibit lower VD and PD compared to MR and controls, and that these OCTA parameters are associated with increased CV event risk at 5 and 10 years. FAZ metrics, particularly FAZa and FAZp, were strongly associated with the VHR group when comparing adjusted values with HR (Table 4). FAZc was significantly lower in most comparisons between CV risk groups and controls. Regarding structural OCT parameters, no clinically or statistically significant differences were observed in CMT and RNFL after adjustment for age, sex, axial length, DM duration, SSI, and CMT.

The analysis of correlations between current and historical CV risk and OCTA metrics in people with T1D revealed significant findings. A strong inverse correlation was observed between CV risk probability at 5 and 10 years and VD and PD (*p* < 0.001 and *p* < 0.05, respectively), indicating that lower VD and PD values are associated with increased likelihood of fatal or non-fatal CV events. In contrast, no significant associations were found for FAZ metrics (FAZa, FAZp, FAZc), suggesting that FAZ may be a less sensitive predictor of CV risk in the T1D population. The structural OCT parameter CMT showed a significant positive correlation with CV event risk at both 5 and 10 years (*p* < 0.05 for all). There are several potential factors which may contribute to explaining these correlations. Previous reports have described greater retinal thickness in eyes with higher HbA1c levels in the absence of DR, suggesting that a subclinical inflammation may precede the appearance of clinical DR [21]. This worse glycemic control could also be reflected in the CV risk grade. Moreover, CMT is one of the factors which has shown intergroup differences for MR with respect to HR and HVR, with other factors such as age, duration of disease, etc., which could also play a role in the observed findings.

Our findings demonstrate strong associations between OCTA metrics and CV risk probability in people with T1D, reinforcing prior evidence supporting OCTA as a valuable tool for detecting and quantifying microvascular alterations in DM. Reductions in SCP VD and PD, along with FAZ enlargement, may occur even in early, subclinical stages of DR and are correlated with both DR severity and VA [22,23]. Kim et al. conducted a retrospective cross-sectional study comparing 84 eyes with DR to 14 healthy controls, showing that increasing DR severity was associated with significant declines in VD, skeleton density, and fractal dimension, and a positive correlation with vessel diameter index (*p* < 0.001) [24]. With regard to CV risk, it should be noted that DR is a target-organ disease, being one of the items that define the VHR group (together with proteinuria, with renal impairment defined as eGFR < 30 mL/min/1.73 m^2^ or left ventricular hypertrophy, or a combination of three of age, hypertension, dyslipidemia, smoking and/or obesity). As a consequence, all DR patients were classified in the VHR group. Due to the multiple correlation pathways of DM, it is not possible to isolate the role of this factor on OCTA metrics in the VHR group, but at the same time it highlights the role of OCTA in non-DR patients (classified in MR and HR groups). These findings underscore the potential of OCTA metrics as a non-invasive tool for CV risk stratification in people with T1D, which could ultimately support clinicians in identifying patients which potentially could benefit from early interventions to reduce progression to adverse CV outcomes.

It is also important to note that various CV risk factors may influence OCTA measurements and introduce potential bias in the detection and quantification of DM-related disorders. Monteiro-Henriques et al. reported that common CV risk factors such as hypertension (HTN), renal disease, pre-eclampsia, coronary artery disease, carotid artery stenosis, obstructive sleep apnea, and DM, are generally associated with reduced retinal and choroidal VD and vessel length, as well as increased FAZa and FAZp [8]. Additionally, several characteristics or conditions, including race, are linked to retinal microvascular changes and should be considered. Other studies have found significant correlations between OCTA metrics and smoking status, BMI, HTN, elevated TG, and higher HbA1c levels in people with T1D [21,25]. The pathophysiology underlying CV events in T1D remains unclear, and the relative contribution of conventional CV risk factors is not yet well defined [13].

Unlike patients with T2D, conventional CV risk factors such as hyperlipidaemia, HTN, and smoking, are rarely present at the time of T1D diagnosis and typically emerge several years later. Currently, no algorithms are routinely used in standardized clinical practice to predict CV risk in people with T1D, and most models developed for healthy individuals or T2D patients have shown poor predictive performance. For instance, the Framingham Heart Study algorithm tends to underestimate CV event risk in T1D, as conventional CV risk factors fail to fully account for the elevated risk, suggesting the presence of T1D-specific contributors [15]. People with T1D often experience long-term hyperglycaemia independent of other metabolic disorders, unlike those with T2D. The ESC guidelines stratify T1D patients into MR, HR, and VHR categories for fatal CV events over 10 years, based primarily on observational data, though these classifications lack validation against actual CV outcomes [2].

Recent studies have evaluated the concordance between ESC CV risk classification and the STENO T1 Risk Engine. Tecce et al. assessed the agreement between the 2019 ESC CV risk categories and 10-year CV risk predictions using the STENO T1 Risk Engine in 575 adults with T1D (mean age 36 ± 12 years) [18]. According to ESC criteria, 45% of patients with T1D without CV disease were classified as VHR. However, none of these were <35 years old, and only 12% of those > 35 years were confirmed as VHR by the STENO algorithm. Serés-Noriega et al. included 501 patients with T1D (mean age 48.8 years; median DM duration 26.5 years), reporting poor agreement between the two tools in identifying preclinical atherosclerosis among HR T1D adults [26]. Both studies suggest that the STENO T1 Risk Engine provides more accurate and individualized CV risk assessment and highlight substantial differences in the initiation and eligibility for blood pressure, cholesterol, and antiplatelet therapy depending on the stratification tool used—potentially impacting clinical management, particularly in younger patients [27].

The emergence of Oculomics has introduced novel opportunities for personalized CV risk stratification, as machine learning (ML) and deep learning (DL) models can extract retinal microvascular biomarkers that reflect systemic CV health. Wenyi Hu et al. conducted a systematic review and meta-analysis of 26 studies applying DL to retinal images to predict various CV disease-related outcomes [28]. Three studies aiming to forecast future CV events reported Area Under Curve (AUC) values ranging from 0.68 to 0.81. Additionally, models using retinal images as input data performed well in predicting individual risk factors such as age (mean absolute error, MAE = 3.19 years), gender, DM, and chronic kidney disease, with AUC values between 0.80 and 0.96. Germanese et al. used retinal images to predict the neurocardiovascular risk score (CHA_2_DS_2_-VASc) from the open-source RASTA dataset of 491 patients [29,30]. ML models achieved high predictive accuracy (AUC up to 0.96), while a DL model based on OCTA images (EfficientNetV2-B3) correctly classified 68% of cases, with an MAE of approximately 0.697. Similarly, we applied a radiomics-based approach to estimate CV risk using ML techniques on multimodal retinal imaging [31]. The model showed good performance, with AUC values of 0.79 (MR vs. HR and VHR), and 0.73 (HR vs. VHR). Performance improved substantially with the inclusion of clinical variables, reaching AUCs of 0.99 and 0.95, respectively. These findings support the potential of Oculomics as a promising tool for CV risk stratification. However, the clinical integration of this technology into a feasible model of care requires careful consideration, as CV risk prediction remains challenging due to its multifactorial and systemic nature [32]. Multimodal AI models combining retinal imaging and minimal clinical data may offer improved predictive performance. Nevertheless, current limitations of ML and DL models for CV risk prediction from retinal images include small, ethnically homogeneous datasets and limited external validation, which restrict generalizability. There is considerable variability in study designs, outcome measures, and imaging protocols, underscoring the need for larger, multi-ethnic, and standardized datasets. Furthermore, prospective studies in real-world clinical settings are essential to validate the practical applicability and accuracy of these models prior to widespread implementation [28].

The strengths of this study include the large sample size, the specific focus on people with T1D, and the collection of high-quality data, as both patients and controls were prospectively enrolled in a clinical trial setting with blood sampling and comprehensive retinal imaging. However, several limitations should be noted. First, the commercial OCT device used only allows quantitative measurements in the SCP, excluding the deep capillary plexus, which has been identified by some authors as the initial site of microvascular damage in DR progression [33]. This limitation could be addressed in future studies using customized research software to investigate the association of OCTA metrics measured in this plexus, which potentially may detect earlier microvascular changes and therefore earlier CV risk grade detection. At the same time, particularly if SCP damage is less pronounced, this consideration may enhance the clinical relevance of the current findings. Alternatively, novel 3D technologies applied to OCTA that enable analysis of the entire macular vascular network could overcome this issue [34,35]. Second, other CV risk factors such as smoking, BMI, HTN, and TG, though less prevalent in T1D, may introduce bias in OCT and OCTA measurements. Third, participants who consented to join the study may represent a subgroup of healthier, more adherent and more regularly screened individuals with T1DM compared to the general population. Fourth, as previously mentioned, DR is one of the items that define the VHR group, making it impossible to isolate the influence of this parameter in OCTA metrics in this specific group. At the same time, it adds relevance to OCTA metrics in non-DR patients (MR and HR groups). Finally, some of the adjusted correlations for age, sex, axial length, SSI, and DM duration using the STENO T1 Risk Engine revealed no significant associations between OCT/OCTA metrics and CV risk, suggesting that additional factors may contribute to ocular vascular alterations and should be considered.

## 5. Conclusions

This study specifically investigates CV risk profiles and OCT/OCTA parameters as potential markers of systemic disease status in T1D, suggesting that these retinal imaging techniques could be integrated into routine clinical care during annual check-ups. These objective measurements could serve as useful tools for CV risk profiling and potentially have a direct impact on the systemic management of T1D patients in the future. In summary, retinal microvasculature analysis via OCTA, a rapid, objective, and non-invasive test, holds promise as a tool for systemic CV risk assessment in targeted risk groups or population-based screening programs, potentially serving as a valuable CV risk biomarker that could be incorporated into diagnostic algorithms for prevalent CV disease in the near future.

## Figures and Tables

**Table 1:** Baseline characteristics of the study cohort. Demographics and diabetes-related clinical characteristics. Comparisons between study subgroups: Control–Moderate Risk (a), Control–High Risk (b), Control–Very High Risk (c), Moderate Risk–High Risk (d), Moderate Risk–Very High Risk (e), High Risk–Very High Risk (f).

Variable	Number of Eyes (C/M/H/VH)	Statistics	Control	Moderate Risk	High Risk	Very High Risk	*p*-Value
Demographics							
Age (years)	(104/37/152/208)	Mean (SD)	43.35 (14.25)	28.13 (5.83)	38.18 (12.06)	42.37 (11.17)	a, b, d, e, f
		Median (Q_1_, Q_3_)	41.10 (30.98, 56.35)	27.10 (23.70, 33.10)	37.35 (28.28, 46.73)	41.55 (34.15, 50.00)	a, b, d, e, f
Sex, female	(104/37/152/208)	*n* (%)	63 (60.6%)	15 (40.5%)	81 (53.3%)	104 (50.0%)	-
Smoking habits	(98/37/152/208)						
*Nonsmoker*		*n* (%)	71 (72.4%)	37 (100.0%)	95 (62.5%)	113 (54.3%)	
*Actual smoker*		*n* (%)	8 (8.2%)	0 (0.0%)	37 (24.3%)	49 (23.6%)	a, b, c, d, e
*Ex-smoker*		*n* (%)	19 (19.4%)	0 (0.0%)	20 (13.2%)	46 (22.1%)	
Hypertension	(97/37/152/208)	*n* (%)	9 (9.3%)	0 (0.0%)	5 (3.3%)	36 (17.3%)	e, f
BMI (kg/m^2^)	(95/37/151/208)	Mean (SD)	23.64 (3.50)	22.96 (2.58)	24.29 (3.71)	25.42 (3.88)	c, d, e, f
		Median (Q_1_, Q_3_)	23.18 (21.20, 25.46)	22.84 (21.09, 24.08)	23.67 (21.59, 26.79)	24.91 (22.72, 27.70)	c, e, f
Diabetes-related clinical characteristics							
DM duration (years)	(-/37/152/206)	Mean (SD)	-	5.58 (2.75)	15.79 (7.95)	25.63 (9.05)	d, e, f
		Median (Q_1_, Q_3_)	-	6.00 (2.60, 8.20)	16.05 (10.28, 20.10)	25.65 (20.02, 31.70)	d, e, f
Macrovascular complications	(98/37/152/207)						
*Cerebrovascular disease*		*n* (%)	0 (0.0%)	0 (0.0%)	0 (0.0%)	4 (1.9%)	-
*Ischemic heart disease*		*n* (%)	1 (1.0%)	0 (0.0%)	0 (0.0%)	4 (1.9%)	-
*Peripheral vascular disease*		*n* (%)	1 (1.0%)	0 (0.0%)	0 (0.0%)	2 (1.0%)	-
Insulin requirements (UI/kg)	(-/37/150/208)	Mean (SD)	-	0.60 (0.23)	0.62 (0.24)	0.64 (0.25)	-
		Median (Q_1_, Q_3_)	-	0.60 (0.41, 0.79)	0.60 (0.45, 0.76)	0.63 (0.50, 0.80)	-

**Table 2:** Baseline characteristics of the study cohort. Laboratory tests. Comparisons between study subgroups: Control–Moderate Risk (a), Control–High Risk (b), Control–Very High Risk (c), Moderate Risk–High Risk (d), Moderate Risk–Very High Risk (e), High Risk–Very High Risk (f).

Variable	Number of Eyes (C/M/H/VH)	Statistics	Control	Moderate Risk	High Risk	Very High Risk	*p*-Value
Laboratory tests							
HbA1c (%)	(72/37/151/195)	Mean (SD)	5.37 (0.33)	7.05 (0.86)	7.45 (0.94)	7.53 (0.92)	a, b, c, d, e
		Median (Q_1_, Q_3_)	5.35 (5.18, 5.60)	6.90 (6.50, 7.60)	7.40 (6.85, 7.85)	7.40 (6.90, 8.10)	a, b, c, d, e
Total cholesterol (mg/dL)	(72/37/150/198)	Mean (SD)	194.42 (31.90)	157.76 (23.30)	176.80 (28.66)	179.53 (31.68)	a, b, c, d, e
		Median (Q_1_, Q_3_)	195.00 (173.0, 215.25)	160.00 (142.00, 176.0)	176.50 (158.00, 193.0)	178.00 (156.00, 199.0)	a, b, c, d, e
LDL cholesterol (mg/dL)	(72/37/150/186)	Mean (SD)	115.69 (30.63)	90.54 (19.65)	100.50 (25.07)	104.15 (23.82)	a, b, c, d, e
		Median (Q_1_, Q_3_)	114.50 (93.50, 140.00)	89.00 (79.00, 105.00)	97.50 (83.00, 117.00)	103.00 (88.25, 120.00)	a, b, c, d, e
HDL cholesterol (mg/dL)	(72/37/150/196)	Mean (SD)	57.01 (13.83)	55.11 (14.69)	61.18 (16.81)	58.32 (18.62)	d
		Median (Q_1_, Q_3_)	57.00 (48.75, 67.00)	53.00 (46.00, 62.00)	58.00 (49.00, 71.75)	55.00 (45.00, 69.00)	-
Triglycerides (md/dL)	(72/37/150/199)	Mean (SD)	113.38 (57.57)	60.68 (19.92)	75.88 (33.20)	90.43 (61.89)	a, b, c, d, e, f
		Median (Q_1_, Q_3_)	103.50 (66.75, 141.75)	55.00 (49.00, 72.00)	71.00 (53.25, 89.00)	72.00 (56.00, 101.50)	a, b, c, d, e
Hemoglobin (g/L)	(72/34/143/185)	Mean (SD)	136.89 (12.50)	143.82 (13.43)	141.03 (11.86)	142.32 (13.09)	a, b, c
		Median (Q_1_, Q_3_)	135.50 (129.00, 144.0)	145.00 (134.25, 153.0)	141.00 (132.00, 149.5)	142.00 (134.00, 152.0)	a, b, c
Platelets (10^9^/L)	(72/34/143/185)	Mean (SD)	251.44 (52.53)	238.12 (57.82)	249.01 (59.43)	254.35 (57.86)	-
		Median (Q_1_, Q_3_)	251.50 (206.75, 296.0)	247.00 (213.5, 261.25)	246.00 (206.00, 285.0)	251.00 (211.0, 292.0)	-

**Table 3:** Baseline characteristics of the study cohort. Ocular characteristics. Comparisons between study subgroups: Control–Moderate Risk (a), Control–High Risk (b), Control–Very High Risk (c), Moderate Risk–High Risk (d), Moderate Risk–Very High Risk (e), High Risk–Very High Risk (f).

Variable	Number of Eyes (C/M/H/VH)	Statistics	Control	Moderate Risk	High Risk	Very High Risk	*p*-Value
Ocular characteristics							
Visual Acuity	(103/37/152/208)	Mean (SD)	84.30 (1.55)	84.38 (1.09)	83.82 (1.82)	83.09 (3.50)	b, c, d, e, f
		Median (Q_1_, Q_3_)	85.00 (84.00, 85.00)	85.00 (84.00, 85.00)	84.00 (84.00, 85.00)	84.00 (83.75, 85.00)	b, c, d, e
Axial Length	(102/37/151/207)	Mean (SD)	23.77 (1.05)	24.05 (1.07)	23.63 (1.19)	23.38 (1.11)	c, d, e, f
		Median (Q_1_, Q_3_)	23.64 (23.02, 24.49)	23.97 (23.30, 24.97)	23.43 (22.87, 24.34)	23.24 (22.68, 23.99)	c, d, e
Spherical Equivalent	(100/36/151/208)	Mean (SD)	−0.38 (1.97)	−1.43 (1.78)	−0.79 (2.11)	−0.51 (2.21)	a, e
		Median (Q_1_, Q_3_)	−0.25 (−1.28, 0.53)	−1.00 (−2.12, −0.22)	−0.38 (−1.69, 0.25)	−0.38 (−1.62, 0.53)	a, e
Diabetic Retinopathy	(104/37/152/208)						e, f
*No Diabetic Retinopathy*		*n* (%)	-	37 (100.0%)	152 (100.0%)	55 (26.4%)	
*Mild NPDR*		*n* (%)	-	0 (0%)	0 (0%)	128 (61.5%)	
*Moderate NPDR*		*n* (%)	-	0 (0%)	0 (0%)	21 (10.1%)	
*Severe NPDR*		*n* (%)	-	0 (0%)	0 (0%)	2 (1.0%)	
*PDR*		*n* (%)	-	0 (0%)	0 (0%)	2 (1.0%)	

**Table 4:** Subgroup analysis based on Cardiovascular risk stratification. Comparisons between study subgroups: Control–Moderate Risk (a), Control–High Risk (b), Control–Very High Risk (c), Moderate Risk–High Risk (d), Moderate Risk–Very High Risk (e), High Risk–Very High Risk (f).

Variable	N Eyes (C/M/H/VH)	Statistics	Control	Moderate Risk	High Risk	Very High Risk	*p*-Value *0	*p*-Value *1	*p*-Value *2	*p*-Value *3
OCTA 3X3										
Vessel Density (mm^−1^)	(97/36/144/195)	Mean (SD)	20.82 (1.73)	20.99 (1.30)	20.24 (1.67)	19.30 (1.81)	b, c, d, e, f	e, f	b, c, d, e	a, b, c,
		Median (Q_1_, Q_3_)	21.20 (20.10, 22)	21.40 (20.48, 22)	20.55 (19.38, 21.42)	19.50 (18.3, 20.5)	b, c, d, e, f			
Perfusion Density	(97/36/144/195)	Mean (SD)	0.372 (0.029)	0.375 (0.021)	0.366 (0.027)	0.358 (0.028)	c, d, e, f	-	c, d, e, f	a, b, c
		Median (Q_1_, Q_3_)	0.378 (0.363, 0.392)	0.378 (0.364, 0.390)	0.371 (0.351, 0.386)	0.362 (0.345, 0.378)	b, c, e, f			
FAZ Area (mm^2^)	(89/36/127/175)	Mean (SD)	0.230 (0.088)	0.245 (0.078)	0.223 (0.101)	0.249 (0.105)	f	f	c, f	-
		Median (Q_1_, Q_3_)	0.230 (0.17, 0.29)	0.230 (0.208, 0.273)	0.210 (0.160, 0.290)	0.240 (0.17, 0.31)	f			
FAZ Perimeter (mm)	(89/36/127/175)	Mean (SD)	2.034 (0.428)	2.081 (0.335)	1.984 (0.514)	2.159 (0.526)	c, f	f	c, e, f	b
		Median (Q_1_, Q_3_)	2.060 (1.780, 2.34)	2.055 (1.912, 2.215)	1.990 (1.675, 2.3)	2.200 (1.770, 2.5)	c, f			
FAZ Circularity	(89/36/127/175)	Mean (SD)	0.674 (0.075)	0.695 (0.060)	0.652 (0.076)	0.631 (0.096)	b, c, d, e, f	d, e	b, c, d, e	a, d, e
		Median (Q_1_, Q_3_)	0.680 (0.630, 0.73)	0.700 (0.660, 0.732)	0.660 (0.6, 0.7)	0.650 (0.58, 0.7)	b, c, d, e			
OCT Macular										
Central Macular Thickness (μm)	(101/37/148/199)	Mean (SD)	262.604 (22.190)	256.946 (16.347)	264.034 (21.174)	264.216 (22.421)	d, e	-		
		Median (Q_1_, Q_3_)	260 (247, 280)	255 (249, 268)	265.5 (251, 280.2)	264 (249, 277)	d, e			
Macular Volume	(101/37/148/199)	Mean (SD)	10.288 (0.509)	10.257 (0.438)	10.293 (0.496)	10.281 (0.462)	-	-		
		Median (Q_1_, Q_3_)	10.200 (9.9, 10.6)	10.300 (10.0, 10.5)	10.300 (10.0, 10.6)	10.200 (10.0, 10.6)	-			
Macular Thickness Average (μm)	(101/37/148/199)	Mean (SD)	285.673 (14.088)	284.811 (12.007)	285.831 (13.778)	285.533 (12.764)	-	-		
		Median (Q_1_, Q_3_)	284 (274, 294)	285 (277, 292)	286 (278, 294)	285 (278, 294)	-			
Optic Nerve										
Average RNFL	(96/35/146/192)	Mean (SD)	96.594 (9.048)	93.629 (10.059)	96.219 (10.293)	96.844 (11.034)	-	a, d, e	-	-
		Median (Q_1_, Q_3_)	95.000 (91.000, 104.250)	95.000 (85.000, 101.000)	95.000 (90.000, 101.750)	96.500 (90.750, 104.000)	-			

*0. *p* values unadjusted. *1. *p* values adjusted by age, sex, axial length, DM duration, SSI and CMT. *2. *p* values adjusted by CMT. *3 *p* values adjusted by age, sex, axial length, DM duration, SSI, CMT, DM duration, HbA1c, albuminuria, DR grade, smoking status, hypertension, systolic BP, BMI, triglycerides, lipid profile (cholesterol, LDL, HDL) and HbA1c.

## Data Availability

The datasets used and/or analyzed during the current study are available from the corresponding author on reasonable request.

## Supplementary Materials

The following supporting information can be downloaded at: https://www.mdpi.com/article/10.3390/biomedicines14010153/s1.

## Abbreviations

The following abbreviations are used in this manuscript (in alphabetical order):

AMT	Average Macular Thickness
BCVA	Best-Corrected Visual Acuity
BMI	Body Mass Index
CMT	Central Macular Thickness
CV	Cardiovascular
DL	Deep Learning
DM	Diabetes Mellitus
DR	Diabetic Retinopathy
ESC	European Society of Cardiology
FAZ	Foveal Avascular Zone
FAZa	Foveal Avascular Zone (area)
FAZc	Foveal Avascular Zone (circularity)
FAZp	Foveal Avascular Zone (perimeter)
HbA1c	Glycated Hemoglobin
HDL	High-Density Lipoprotein
LDL	Low-Density Lipoprotein
ML	Machine Learning
MV	Macular Volume
NICE	National Institute for Health and Care Excellence
OCT	Optical Coherence Tomography
OCTA	Optical Coherence Tomography Angiography
PD	Perfusion Density
RNFL	Retinal Nerve Fiber Layer
SCP	Superficial Capillary Plexus
SSI	Signal Strength Index
T1D	Type 1 Diabetes
T2D	Type 2 Diabetes
VA	Visual Acuity
VD	Vessel Density
