# Supplementary material for: Optical Coherence Tomography Angiography in Type 1 Diabetes Mellitus. Report 5: Cardiovascular Risk
Source: Biomedicines. 2026 Jan 11;14(1):153. doi: 10.3390/biomedicines14010153 (PMC12838605; doi:10.3390/biomedicines14010153)
Supplement: Supplementary file 1 [file biomedicines-14-00153-s001.zip › Supplementary File S1.pdf]

Study power for between-risk-group comparisons

Minimum detectable differences for 80% power (1-β) and 5% significance level (α) in OCTA measurements:

α=0.05 (5%) / 1-β = 0.8 (80%)			
Welch`s t test	Moderate – High Risk	Moderate – Very High Risk	High Risk – Very High Risk
	Hypothesized mean difference between groups		
Vessel Density (mm-1)	0.73	0.72	0.53
Perfusion Density	0.012	0.011	0.0085
FAZ area (mm^2)	0.045	0.043	0.034
FAZ perimeter (mm)	0.204	0.202	0.170
FAZ circularity	0.034	0.035	0.028
