# Supplementary material for: Optical Coherence Tomography Angiography in Type 1 Diabetes Mellitus. Report 5: Cardiovascular Risk
Source: Biomedicines. 2026 Jan 11;14(1):153. doi: 10.3390/biomedicines14010153 (PMC12838605; doi:10.3390/biomedicines14010153)
Supplement: Supplementary file 1 [file biomedicines-14-00153-s001.zip › Supplementary File S2.pdf]

OCT and OCTA scans excluded due to Signal Strenght Index (SSI) <7 per Cardiovascular Risk groups

Excluded Scans	Control (N total = 104)	Moderate Risk (N total = 37)	High Risk (N total = 152)	Very High Risk (N total = 208)	P value (Fisher exact test)
OCTA 3x3	7 (6.7%)	1 (2.7%)	8 (5.3%)	13 (6.3%)	0.909
FAZ OCTA 3x3	15 (14.4%)	1 (2.7%)	25 (16.4%)	33 (15.9%)	0.142
OCTA Macular	3 (2.9%)	0 (0.0%)	4 (2.6%)	9 (4.3%)	0.668
Optic Nerve	8 (7.7%)	2 (5.4%)	6 (3.9%)	16 (7.7%)	0.481
