# Supplementary material for: Optical Coherence Tomography Angiography in Type 1 Diabetes Mellitus. Report 5: Cardiovascular Risk
Source: Biomedicines. 2026 Jan 11;14(1):153. doi: 10.3390/biomedicines14010153 (PMC12838605; doi:10.3390/biomedicines14010153)
Supplement: Supplementary file 1 [file biomedicines-14-00153-s001.zip › Supplementary File S3.pdf]

Bootstrapping

Replacement analysis using 300 bootstrap steps was applied to obtain reliable group comparison´s coefficient distributions and percentile derivate bootstrap p values.

Control-Moderate Risk (a), Control-High Risk (b), Control-Very High Risk (c), Moderate Risk-High Risk (d), Moderate Risk-Very High Risk (e), High Risk-Very High Risk (f)

Bootstrapping with replacement (300 bootstrap steps)	C-M (a)	C-H (b)	C-VH (c)	M-H (d)	M-VH (e)	H-VH (f)	Estimated bootstrapping p value	Paper reported p value
	Bootstrap Estimated Effect Percentile (2.5%/97.5%)							
Vessel densitiy (Welch t test)	-0.611/ 0.406	0.203/ 1.100	1.158/ 1.936	0.250/ 1.215	1.242/ 2.180	0.577/ 1.291	b,c,d,e,f	b,c,d,e,f
Vessel densitiy (Adjusted*)	-0.695/ 0.182	-0.713/ 0.180	-0.285/ 0.719	-0.442/ 0.416	-0.038/ 0.888	0.120/ 0.732	f	f
Perfusion density (Welch t test)	-0.010/ 0.007	-0.002/ 0.013	0.007/ 0.021	0.001/ 0.016	0.009/ 0.024	0.001/ 0.013	c,d,e,f	c,d,e,f
Perfusion density (Adjusted*)	-0.011/ 0.004	-0.011/ 0.004	-0.008/ 0.008	-0.006/ 0.008	-0.004/ 0.012	-0.001/ 0.009	-	-
FAZ area (Welch t test)	-0.047/ 0.016	-0.021/ 0.037	-0.040/ 0.006	-0.012/ 0.055	-0.035/ 0.028	-0.051/ 0.001	-	f
FAZ area (Adjusted*)	-0.063/ 0.006	-0.034/ 0.037	-0.058/ 0.022	-0.001/ 0.063	-0.029/ 0.049	-0.044/ 0.005	-	-
FAZ perimeter	-0.194/ 0.086	-0.074/ 0.156	-0.233/-0.009	-0.018/ 0.236	-0.205/ 0.067	-0.281/-0.055	c,f	c,f

(Welch t test)								
FAZ perimeter (Adjusted*)	-0.231/0.063	-0.143/0.217	-0.298/0.080	-0.020/0.260	-0.217/0.123	-0.277/-0.035	f	f
FAZ circularity (Welch t test)	-0.045/0.001	0.004/0.042	0.025/0.062	0.022/0.067	0.043/0.088	0.003/0.039	b,c,d,e,f	b,c,d,e,f
FAZ circulatiry (Adjusted*)	-0.050/0.000	-0.018/0.034	-0.017/0.047	0.006/0.054	0.010/0.070	-0.010/0.031	d,e	d,e

*Multivariate regression (ANCOVA) adjusted by age, sex, axial length, DM duration and SSI
